# Optimizing Pediatric Chest Compressions: A Randomized Crossover Simulation Trial of Over-the-Head vs. Lateral Techniques

**DOI:** 10.3390/pediatric17020044

**Published:** 2025-04-08

**Authors:** Malgorzata Kietlinska, Wojciech Wieczorek, Michal Pruc, Lukasz Szarpak, Grazyna Nowak-Starz, Wojciech Flieger, Burak Katipoglu, Monika Tomaszewska

**Affiliations:** 1 Department of Clinical Research and Development, LUXMED Group, 02-678 Warsaw, Poland; 2 Department of Emergency Medicine, Medical University of Warsaw, 02-005 Warsaw, Poland; 3 Institute of Medical Science, Collegium Medicum, The John Paul II Catholic University of Lublin, 20-950 Lublin, Poland; 4 Henry JN Taub Department of Emergency Medicine, Baylor College of Medicine, Houston, TX 77030, USA; 5 Collegium Medicum, Jan Kochanowski University, 25-317 Kielce, Poland; 6 Department of Emergency Medicine, Ankara Etlik City Hospital, 06510 Ankara, Turkey

**Keywords:** pediatric cardiac arrest, cardiopulmonary resuscitation, chest compressions, quality, over-the-head technique, lateral technique, simulation study, rescuer fatigue

## Abstract

Background/Objectives: Pediatric cardiac arrest poses considerable obstacles, with survival rates markedly inferior to those of adults. Effective chest compressions are essential for enhancing outcomes; nevertheless, the ideal rescuer attitude is still ambiguous. This study sought to compare the efficacy of lateral (LAT) and over-the-head (OTH) chest compression techniques in pediatric cardiopulmonary resuscitation (CPR) and to ascertain whether OTH presents a viable alternative to the conventional LAT method by assessing compression quality, rescuer fatigue, and ergonomics. Methods: A randomized crossover simulation study was conducted in a high-fidelity medical simulation facility. Thirty-five medical students executed 2 min cycles of chest compressions with both LAT and OTH techniques, interspersed with a 15 min rest period between sessions. Results: OTH showed a tendency for enhanced overall performance (72.94 vs. 64.46; *p* = 0.08), while the differences lacked statistical significance. The compression rate was somewhat elevated with OTH (116.94 compared to 114.57; *p* = 0.31). We assessed LAT as somewhat less challenging (4.37 vs. 3.91; *p* = 0.17) and found less fatigue (4.83 vs. 4.40; *p* = 0.24). Male rescuers and individuals with elevated BMI attained larger compression depths. Age was negatively connected with the ease and efficiency of compressions. Conclusions: Although no statistically significant differences were detected, OTH demonstrated potential for enhanced performance. The anthropometrics of rescuers affected the quality of CPR, highlighting the necessity for tailored training methods. Future investigations should examine the long-term viability of OTH in clinical and pre-hospital environments.

## 1. Introduction

Pediatric out-of-hospital cardiac arrest (OHCA) presents a considerable challenge, as survival rates are significantly lower compared to adults [1]. Epidemiological data indicate that the incidence of pediatric out-of-hospital cardiac arrest (OHCA) is approximately 8 to 10 per 100,000 children annually, with survival to hospital discharge varying from 5% to 10%, contingent upon the underlying cause and the quality of pre-hospital interventions [2,3,4]. In-hospital cardiac arrest (IHCA) among pediatric patients occurs at a rate of 1 to 3 per 1000 hospital admissions, with some studies reporting survival rates of 33.8%. Common etiologies comprise respiratory failure, sepsis, and congenital heart disease [5,6,7]. The etiology of pediatric OHCA significantly differs from that of adults, with pediatric cases more frequently resulting from respiratory failure and congenital abnormalities, whereas adult arrests are predominantly of cardiac origin, which contributes to differing incidence and survival rates [8]. Despite advancements in resuscitation techniques and post-cardiac arrest care, neurological outcomes continue to be a concern, as a considerable proportion of survivors exhibit varying degrees of neurological impairment [9]. Effective cardiopulmonary resuscitation (CPR) is essential in pediatric cardiac arrest, as high-quality chest compressions and timely ventilation significantly influence survival outcomes. Optimizing CPR techniques, particularly rescuer positioning, is essential for enhancing survival and functional outcomes in this vulnerable population. The European Resuscitation Council (ERC) and the American Heart Association (AHA) highlight the significance of proper hand positioning and rescuer ergonomics to achieve effective compressions and reduce rescuer fatigue [10,11]. In pediatric CPR, several chest compression techniques have been suggested, including the standard lateral (LAT) position and the over-the-head (OTH) method, especially in cases with single rescuers.

Recent studies have examined the feasibility and efficacy of various compression positions in neonatal and infant cardiopulmonary resuscitation (CPR). Aranda-García et al. performed a randomized simulation study to compare the OTH and LAT positions in infant CPR executed by lifeguards [12]. The findings demonstrated similar overall CPR quality between the two techniques, with the OTH method showing potential advantages in achieving optimal chest recoil, indicating its applicability in specific scenarios. Cheung et al. examined CPR quality in neonatal resuscitation and observed no significant differences between OTH and LAT positioning, supporting the potential interchangeability of these methods [13]. Jo et al. demonstrated that the OTH two-thumb encircling technique resulted in greater compression depth and improved rescuer ergonomics compared to the traditional two-finger technique in in-hospital infant CPR settings [14,15].

Despite these insights, a gap persists in the literature concerning the comparative effectiveness of OTH versus LAT positioning in pediatric chest compressions beyond neonatal resuscitation. Although research has mainly concentrated on simulation-based evaluations, further investigation is necessary regarding the effects of rescuer fatigue and the correction of hand placement in pediatric populations. The potential benefits of OTH positioning in pre-hospital and resource-limited settings, where space constraints or single-rescuer scenarios may require alternative CPR techniques, merit further investigation.

This study compares the efficacy of lateral versus over-the-head chest compression techniques in pediatric CPR, focusing on compression depth, rate, chest recoil, and rescuer fatigue. By assessing these parameters, we aimed to determine whether OTH offers a viable alternative to the traditional LAT approach, potentially informing CPR guidelines and training protocols.

## 2. Materials and Methods

This research employed a randomized crossover design to assess the efficacy and performance of two juvenile chest compression techniques: the traditional LAT position and the OTH method. The inquiry was performed in a high-fidelity medical simulation facility, guaranteeing a controlled setting that accurately mirrored real-world pediatric resuscitation situations. Ethical approval was secured by the Institutional Review Board of the Polish Society of Disaster Medicine (IRB No. 45-2024-0312-IRB), and the study complied rigorously with worldwide ethical standards and best practices in clinical research.

### 2.1. Participant Selection

Eligible participants consisted of medical students currently enrolled in AHA-accredited Pediatric Advanced Life Support (PALS) courses. Although participants had completed a certified Pediatric Advanced Life Support (PALS) course, they had no prior clinical experience in delivering cardiopulmonary resuscitation in real-life settings, ensuring that their performance reflected simulation-based training rather than clinical exposure. To establish a uniform baseline of resuscitation competence, the inclusion criteria mandated prior certification in Basic Life Support (BLS) [3]. Participants having a history of upper-limb injuries in the preceding six months or those without prior pediatric CPR training were excluded to mitigate potential confounding variables associated with physical restrictions or performance discrepancies.

### 2.2. Study Protocol

**Figure 1:**
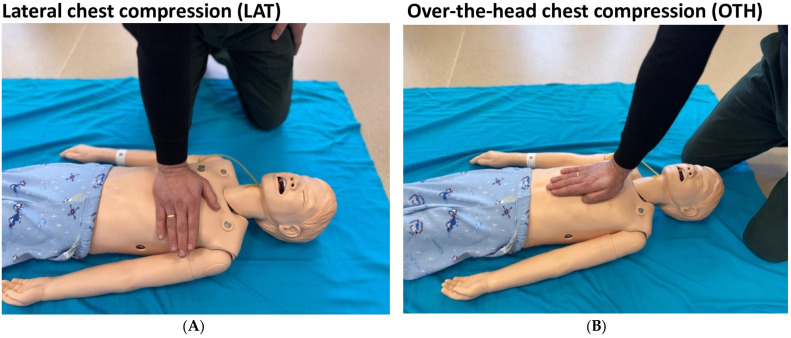
Chest compression techniques: (**A**) lateral (LAT) position, with the rescuer positioned beside the manikin; (**B**) over-the-head (OTH) position, with the rescuer over the head of the manikin delivering vertical compressions.

Participants underwent a standardized pediatric resuscitation curriculum, commencing with a 60 min didactic module that addressed cardiac arrest pathophysiology, revised CPR protocols, and the technical execution of both LAT and OTH techniques (Figure 1).

**Figure 2:**
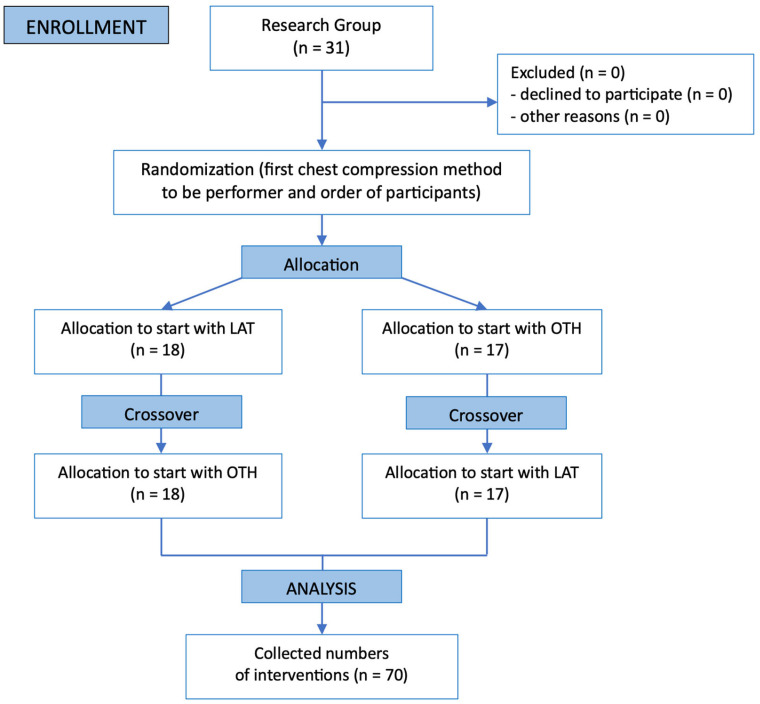
Randomization flow chart.

A supervised practical training module, using a high-fidelity pediatric manikin (MegaCode Kid; Laerdal Medical, Stavanger, Norway), followed the theoretical session. To illustrate the techniques, Figure 2 presents photographs of the LAT and OTH positions as performed during the simulation. In the LAT position, the rescuer is positioned laterally to the manikin with hands placed on the sternum, while in the OTH position, the rescuer stands at the head of the manikin, delivering compressions vertically over the chest. We employed a distinct simulation system (SimJunior^®^; Laerdal Medical, Norway) for data acquisition to mitigate potential training bias. To ensure measurement accuracy, the manikin was calibrated before the experiment by placing it on a stable surface under controlled lighting conditions. Block randomization allocated participants to initiate compressions with either LAT or OTH (Figure 2). The experimental procedure was conducted as follows: (1) Preparation Phase: The high-fidelity manikin (SimJunior^®^; Laerdal Medical, Norway) was calibrated on a stable surface with controlled lighting, and participants were briefed on the assigned technique (LAT or OTH). (2) Execution Phase: Participants performed a 2 min cycle of continuous chest compressions, maintaining the posture depicted in Figure X—lateral to the manikin for LAT or at the head for OTH—followed by a switch to the alternate technique after the rest period. (3) Rest Phase: A 15 min recovery interval was provided between sessions to minimize fatigue carryover. Each participant executed continuous 2 min cycles of chest compressions, succeeded by a 15 min recovery interval to alleviate fatigue effects.

The crossover approach facilitated direct intra-individual comparisons of technique efficacy while accounting for differences in skill levels. To ensure experimental control, block randomization determined the initial technique (LAT or OTH), and the crossover design allowed each participant to serve as their own control, reducing inter-individual variability. The simulation environment, including manikin settings and lighting, was standardized across all trials. Real-time feedback from the simulator’s integrated system ensured consistent and objective measurement of performance metrics for both groups.

### 2.3. Outcomes

CPR performance was evaluated using a combination of objective quality metrics and subjective rescuer feedback to compare the LAT and OTH techniques. The evaluation process focused on (1) assessing CPR quality metrics, including compression rate, depth, chest recoil, and hand placement precision, alongside subjective measures of exertion and discomfort; (2) involving medical students who performed CPR on a high-fidelity manikin, with objective data captured via the simulator’s feedback system and subjective data self-reported using standardized scales; and (3) examining differences in performance efficiency and rescuer experience between the LAT and OTH techniques.

The assessment of CPR performance utilized both objective and subjective metrics to deliver a thorough evaluation of method efficacy. The principal outcomes concentrated on CPR quality metrics, encompassing compression rate, compression depth, chest recoil, and hand placement precision. The compression rate was evaluated by assessing the mean compressions per minute (CPM) and the proportion of compressions within the advised range of 100–120 CPM. The compression depth was evaluated by determining the average depth in millimeters and the percentage of compressions reaching a minimum depth of 40 mm, in compliance with AHA criteria. Furthermore, chest recoil was assessed by the percentage of compressions attaining complete decompression, while hand placement accuracy was evaluated in accordance with AHA recommendations.

Subjective evaluations comprised the Borg Rating of Perceived Exertion (RPE) to gauge perceived exertion and a 10-point Numerical Rating Scale (NRS) to assess discomfort levels, with 1 denoting the optimal outcome (minimal exertion or discomfort) and 10 signifying the most adverse outcome (maximum exertion or discomfort). A composite score was created to combine both objective performance data and subjective exertion assessments, facilitating a comprehensive evaluation of CPR effectiveness. The simulator’s integrated feedback system facilitated real-time data capture, guaranteeing accurate and impartial measurement of all performance characteristics and improving the reliability of the evaluation process.

### 2.4. Power Analysis and Sample Size

The estimation of sample size was informed by previous crossover studies of pediatric chest compression methods. A power analysis conducted with GPower 3.1 for a within-subject ANOVA (α = 0.05, power = 80%, effect size f = 0.25) indicated that at least 30 participants are necessary to identify statistically significant differences in compression depth, anticipating a mean difference (Δ) of 3.0 mm and a standard deviation (SD) of 2.5 mm [15]. The crossover design made the statistics more accurate by lowering the sample size needed while keeping the analytical precision. This was possible because each participant was their own control, which reduced variation between individuals and improved the estimation of effect size.

### 2.5. Statistical Analysis

All statistical analyses were performed utilizing RStudio (Version 2024.12.0+467; R Foundation for Statistical Computing, Vienna, Austria). The normality of continuous data was evaluated using the Shapiro–Wilk test and visual examination of Q-Q plots. Data were presented as means with standard deviations (SDs) for normally distributed variables and as medians with interquartile ranges (IQRs) for non-normally distributed variables. Categorical variables were represented as counts and percentages.

We used independent t-tests for regularly distributed continuous variables in between-group comparisons and the Mann–Whitney U test for non-normally distributed data. Categorical variables were analyzed using the chi-square test or Fisher’s exact test in cases of low anticipated frequencies. For the comparison of different groups, one-way ANOVA with Tukey’s post hoc test was utilized for normally distributed data, while the Kruskal–Wallis test with Dunn’s post hoc correction was used for non-parametric data.

To evaluate the relationships between demographic and anthropometric characteristics (age, sex, weight, height, and BMI) and chest compression performance parameters for technique B, Pearson’s correlation coefficient (r) was utilized for normally distributed continuous variables, whereas Spearman’s rank correlation coefficient (ρ) was employed for non-normally distributed data. Prior to correlation analysis, categorical variables, including sex, were numerically encoded.

A correlation matrix was created to illustrate the strength and direction of relationships between independent and dependent variables. The significance of correlation was assessed using *p*-values, with *p* < 0.05 deemed statistically significant. Pairwise deletion was employed to address missing data, hence maximizing data availability for each correlation analysis.

All statistical tests were conducted as two-tailed, and the results were analyzed in relation to the current literature on chest compression biomechanics. Data visualization, including scatter plots and correlation heatmaps, was executed utilizing the ‘ggplot2’ and ‘corrplot’ packages in R.

## 3. Results

The study cohort comprised 35 participants, of which 40% were men. The age (years) ranged from 21.1 (2.0). The average body weight (kg) of the participants was 70.2 (15.1), and the average height (cm) was 171.8 (9.5). The participants had a mean Body Mass Index (BMI) of 23.7 (4.4) kg/m^2^, which reflected a significant difference across the participants.

### 3.1. Results of Chest Compressions and Fatigue Outcomes

**Figure 3:**
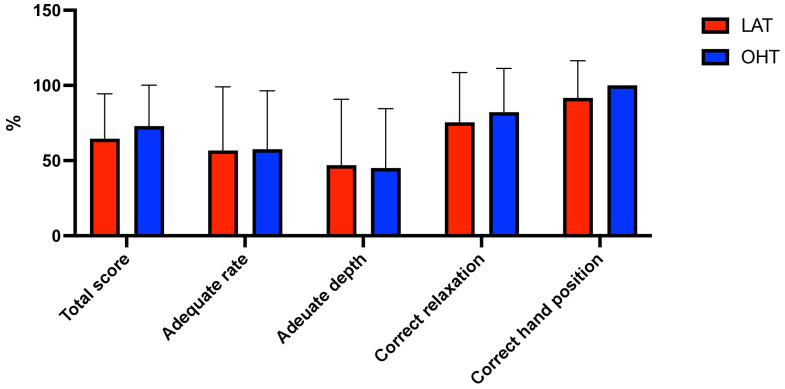
Chest compression quality among research groups. (Legend: CC = chest compression; LAT = lateral position; OHT = over-the-head position).

**Figure 4:**
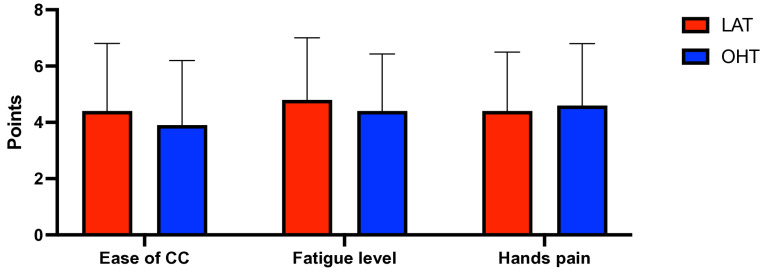
Effect of compression on fatigue and pain complaints. (Legend: LAT = lateral position; OHT = over-the-head position).

To evaluate the differences in performance parameters, a comparative analysis of two chest compressing techniques (LAT and OTH) was performed (Table 1). OTH demonstrated a higher overall performance score (72.94 vs. 64.46), with a trend toward significance (*p* = 0.08), suggesting a potential advantage (Figure 3). Compression frequency was slightly higher in Technique B (116.94 vs. 114.57; *p* = 0.31). LAT was rated as slightly easier (4.37 vs. 3.91; *p* = 0.17) and induced less fatigue (4.83 vs. 4.40; *p* = 0.24). Hand pain was marginally greater in OTH (4.57 vs. 4.43; *p* = 0.67), but differences were not statistically significant (Figure 4).

### 3.2. Relationship Between Performance of Chest Compressions and Anthropometric Variables

A correlation analysis looked into the association of demographic factors (age, gender, weight, height, and BMI) with chest compression performance using both techniques (LAT and OTH).

**Figure 5:**
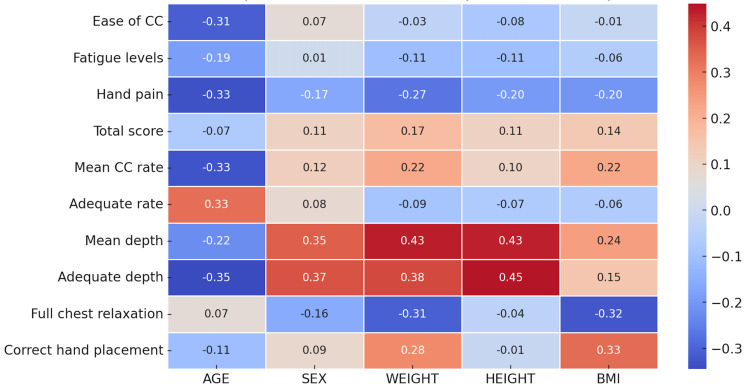
Heatmap of correlations for compression using lateral position technique.

In the LAT technique, age was negatively correlated with ease of compression (r = −0.31) and mean compression rate (r = −0.33), meaning older participants found compressions more difficult and experienced greater discomfort (Figure 5). Male sex had a positive correlation with compression depth (r = 0.35) and correct depth of compression (r = 0.37), which indicates that males were able to achieve deeper compressions. BMI had positive weak correlations with compression depth (r = 0.24) and correct hand position (r = 0.33), and weight had a moderate correlation with compression depth (r = 0.43).

**Figure 6:**
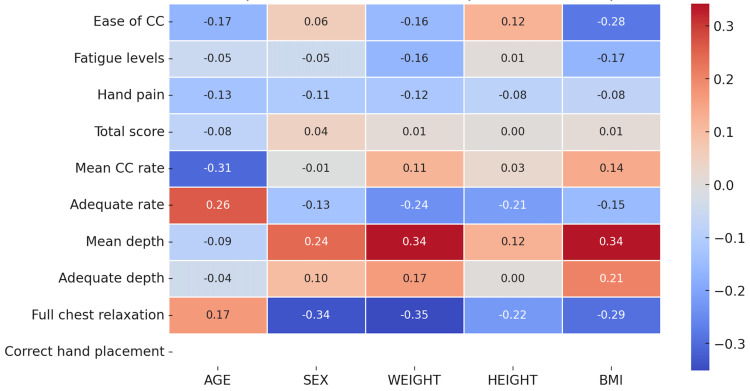
Heatmap of correlations for compression using over-the-head position technique.

In the OTH technique, age had a moderate negative correlation with mean compression rate (r = −0.31) and a weaker correlation with ease of compressions (r = −0.17; Figure 6). Weight had a positive correlation with compression depth (r = 0.34) and correct depth of compression (r = 0.17), while sex had a weak positive correlation with compression depth (r = 0.24). Higher BMI was correlated with greater difficulty and exertion with compressions due to the weak negative correlation with ease of compression (r = −0.28) and tiredness (r = −0.17).

## 4. Discussion

This study offers a comparative analysis of LAT and OTH chest compression techniques in pediatric CPR, highlighting the relationship between technique efficacy, rescuer anthropometrics, and ergonomic factors. Both methods demonstrated feasibility; however, the OTH technique exhibited a trend toward superior overall performance (72.94 vs. 64.46; *p* = 0.08), consistent with the existing literature on optimized CPR delivery in constrained scenarios [16,17]. While this difference did not achieve statistical significance, it is consistent with prior research indicating that OTH may enhance CPR efficiency, especially in situations where quick shifts between chest compressions and ventilations are necessary. Additional factors, including rescuer fatigue, anthropometric variations, and ergonomic strain, should be considered when determining the optimal CPR technique.

The OTH technique produced a marginally elevated compression frequency, averaging 116.94 compressions per minute compared to 114.57 compressions per minute for LAT. This minor difference corresponds with prior research suggesting that OTH improves compression consistency by minimizing interruptions [17]. A 2019 simulation study indicated that the OTH method reduced cycle interruptions by 20% relative to LAT, leading to enhancements in perfusion-critical metrics such as compression depth and rate [17]. This supports our observation that OTH may enhance workflow efficiency, especially during rapid transitions between compressions and ventilations. The LAT technique demonstrated a higher ease of performance (4.37 compared to 3.91) and resulted in reduced rescuer fatigue (4.83 versus 4.40), highlighting a trade-off between technical efficacy and ergonomic sustainability. The findings align with research highlighting the biomechanical strain associated with non-standard CPR positions, potentially undermining extended resuscitation efforts [17,18]. Research on neonatal resuscitation indicates that the sustainability of techniques significantly affects the quality of compressions over time, with ergonomic strain associated with a swift decrease in the delivery of adequate depth [18]. Therefore, although OTH may provide immediate benefits, the ergonomic advantages of LAT may be essential in extended pediatric resuscitation situations where the endurance of the rescuer is crucial.

Anthropometric factors significantly influence CPR effectiveness, as varying physical characteristics affect the capacity to produce adequate compression force. This study identified a positive correlation between male sex and higher body weight and deeper compressions in the LAT technique (r = 0.35 and r = 0.43, respectively). The findings align with prior research indicating that increased upper-body strength and mass enhance force generation during compressions. A 2024 community-wide trial on pediatric CPR performance indicated that children aged 11–14 with a higher body weight (cut-off: 44.8 kg) achieved deeper compressions, with males demonstrating superior performance compared to females in both compression depth and adequate compression fraction [19]. In contrast, age showed a negative correlation with the ease of compression execution (r = −0.31 for LAT) and compression rate (r = −0.33 for LAT; r = −0.31 for OTH), suggesting that older rescuers faced increased challenges in sustaining optimal performance [20]. This finding differs from other pediatric studies, which indicate that older children and young adults typically provide more effective compressions owing to greater physical capacity. The discrepancy may arise from the specific biomechanical requirements of the LAT technique, which necessitates considerable torso stabilization, potentially challenging older rescuers with reduced flexibility or elevated BMI values. The weak negative correlation between BMI and ease of OTH compressions (r = −0.28) indicates that individuals with higher BMI may experience difficulties in applying the vertical force necessary for OTH, potentially due to limited shoulder mobility or heightened fatigue. It is important to note that although participants had completed formal PALS training, their lack of real-world clinical experience may have influenced their CPR performance, particularly in terms of ergonomic adaptation and fatigue management, as demonstrated in previous studies [21,22].

Considering the intricate relationship between compression techniques and rescuer anthropometrics, CPR training must be customized to reflect individual variations in physical capability. Although OTH may enhance compression effectiveness in certain contexts, its efficacy depends on the strength and endurance of the rescuer [23]. Rescuers with lower upper-body strength, including many female practitioners, may require specific strength training to attain sufficient compression depth in both techniques. The ergonomic strain linked to OTH may restrict its use in resource-limited environments or extended resuscitation efforts, where rescuer fatigue is a significant issue. The findings underscore the necessity for adaptive CPR protocols in pediatric care, akin to the European Resuscitation Council’s methodology for neonatal resuscitation, which distinguishes between two-thumb and two-finger techniques [24]. Incorporating anthropometric assessments into tailored training programs may improve preparedness and the overall quality of CPR. Simulation-based CPR training presents a valuable opportunity for enhancing technique selection and optimization. Future research should investigate the incorporation of anthropometric screening tools into training modules, enabling trainees to obtain personalized recommendations for technique selection based on their physical characteristics [25,26]. This approach may enhance technique adoption and improve the effectiveness of resuscitation efforts in real-world emergencies.

Although this study provides valuable insights, it is important to acknowledge several limitations. The simulated CPR environment may not accurately reflect the physiological and psychological stressors encountered in actual resuscitations, which could result in inflated performance scores. The limited sample size and marginal statistical significance of OTH’s advantage (*p* = 0.08) underscore the necessity for validation via larger, multicenter trials. A significant limitation was the absence of ventilation quality metrics, which hindered a thorough assessment of OTH’s claimed efficiency in combining compressions with ventilations. Future research must address these limitations by broadening participant recruitment to include diverse age groups and body compositions, thereby enabling a more accurate assessment of anthropometric influences on CPR performance. Longitudinal studies investigating fatigue-related compression decay are essential for assessing the ability of rescuers to maintain effective compressions over time. Additionally, biomechanical modeling studies must examine force distribution and joint kinematics to determine the most ergonomically efficient compression techniques. Finally, research that incorporates assessments of ventilation quality may clarify whether OTH’s efficiency in workflow transitions leads to enhanced overall resuscitation outcomes.

## 5. Conclusions

No statistically significant differences were seen between the two chest compression techniques; nevertheless, OTH demonstrated a propensity for improved overall performance. Age showed an inverse relationship with the ease, speed, and comfort of compression, although male subjects achieved greater compression depths. An increased BMI was correlated with heightened weariness and perceived challenges, particularly in OTH. This study emphasizes the influence of individual physiological characteristics on CPR performance. Tailored training strategies may enhance effectiveness, particularly for individuals under heightened physical stress. Future research should focus on improving compression techniques based on demographic characteristics to increase both CPR effectiveness and responder endurance.

## Figures and Tables

**Table 1:** Chest compression quality and fatigue outcomes.

Parameter	The Standard Lateral (LAT) Position	The Over-the-Head (OTH) Position	*p*-Value
Total score	64.5 (29.9)	72.9 (27.3)	0.218
Mean rate	114.6 (15.7)	116.9 (11.1)	0.467
Adequate rate (100–120), %	56.7 (42.4)	57.5 (38.9)	0.930
Mean depth, mm	48.1 (7.1)	48.5 (6.4)	0.805
Adequate depth, %	46.9 (43.9)	45.1 (39.4)	0.850
Full relaxation, %	75.4 (33.2)	82.2 (29.1)	0.361
Correct hand position, %	91.7 (24.7)	100.0 (0.0)	0.054
Ease of chest compression	4.4 (2.4)	3.9 (2.3)	0.415
Fatigue level	4.8 (2.2)	4.4 (2.03)	0.400
Hand pain	4.4 (2.1)	4.6 (2.2)	0.782

## Data Availability

The data that support the findings of this study are available on request from the corresponding author (L.S.).

## Abbreviations

The following abbreviations are used in this manuscript:

AHA	American Heart Association
ANOVA	Analysis of Variance
BLS	Basic Life Support
BMI	Body Mass Index
CPM	Compression per Minute
CPR	Cardiopulmonary Resuscitation
ERC	European Resuscitation Council
IHCA	In-Hospital Cardiac Arrest
IQR	Interquartile Range
LAT	Lateral Chest Compression Position
NRS	Numeric Rating Scale
OHCA	Out-of-Hospital Cardiac Arrest
OTH	Over-the-Head Chest Compression Position
PALS	Pediatric Advanced Life Support
SD	Standard Deviation
